# Clinical utility of proteinase 3-antineutrophil cytoplasmic antibody at diagnosis in predicting subsequent relapse in patients with microscopic polyangiitis

**DOI:** 10.3389/fmed.2025.1745280

**Published:** 2026-01-12

**Authors:** Jang Woo Ha, Oh Chan Kwon, Yong-Beom Park, Sang-Won Lee

**Affiliations:** 1 Division of Rheumatology, Department of Internal Medicine, Yongin Severance Hospital, Yonsei University College of Medicine, Yongin, Republic of Korea; 2 Division of Rheumatology, Department of Internal Medicine, Gangnam Severance Hospital, Yonsei University College of Medicine, Seoul, Republic of Korea; 3 Division of Rheumatology, Department of Internal Medicine, Yonsei University College of Medicine, Seoul, Republic of Korea; 4 Institute for Immunology and Immunological Diseases, Yonsei University College of Medicine, Seoul, Republic of Korea

**Keywords:** antibodies, antineutrophil cytoplasmic, microscopic polyangiitis, predict, proteinase 3, recurrence

## Abstract

**Background:**

In real clinical practice, patients classified as microscopic polyangiitis (MPA) according to the new 2022 criteria, despite proteinase 3 (PR3)-antineutrophil cytoplasmic antibody (ANCA) positivity, are occasionally encountered. Hence, this study investigated the clinical utility of PR3-ANCA detected at diagnosis in MPA patients.

**Methods:**

We included 191 patients with MPA in this study and retrospectively reviewed their medical records, in which clinical data at diagnosis and during follow-up were recorded. Additionally, we investigated three major poor complications of MPA, such as subsequent all-cause mortality (ACM), relapse, and end-stage kidney disease (ESKD). Cumulative survival rates were compared among the groups using Kaplan–Meier survival analysis with the log-rank test.

**Results:**

At diagnosis, the median age of 191 MPA patients was 64 years (68 males and 123 females). PR3-ANCA was detected in only 10 patients (5.2%). During follow-up, among the 191 MPA patients, 32 patients died, 43 patients experienced subsequent relapse, and 39 progressed to ESKD. In the comparative analyses, at diagnosis, PR3-ANCA-positive MPA patients were younger and had lower white blood cell count and blood urea nitrogen levels than PR3-ANCA-negative patients. During follow-up, PR3-ANCA-positive MPA patients showed a higher frequency of subsequent relapse than PR3-ANCA-negative patients. Additionally, in Kaplan-Meier survival analysis, PR3-ANCA-positive MPA patients exhibited a significantly lower relapse-free survival rate than PR3-ANCA-negative patients over the follow-up period.

**Conclusion:**

In this study, we found that PR3-ANCA positivity at diagnosis is significantly associated with subsequent relapse in patients with MPA during follow-up.

## 1 Introduction

Antineutrophil cytoplasmic antibody (ANCA)-associated vasculitis (AAV) is one of the two groups of small vessel vasculitides and is characterized by fibrinoid necrotizing vasculitis with few or no immune deposits on histology. AAV is currently composed of three subtypes: microscopic polyangiitis (MPA), granulomatosis with polyangiitis (GPA), and eosinophilic granulomatosis with polyangiitis (EGPA) (1). Each subtype has distinctive clinical, laboratory, radiological, and histopathologic features, but occasionally may also share not a few clinical symptoms with other subtypes (2, 3). For these reasons, in a small group of patients, it might not be possible to clearly define the boundaries between AAV subtypes. Conversely, although it cannot be absolute, ANCA type, such as myeloperoxidase (MPO)-ANCA and proteinase 3 (PR3)-ANCA, could have been helpful for the identification and classification of AAV subtype in such situations (4).

In the algorithm for AAV proposed by the European Medicines Agency in 2007 (the 2007 EMA algorithm), an attempt had been made to classify AAV using the item of any ANCA positivity, instead of distinguishing between MPO-ANCA and PR3-ANCA (5). However, given the differences in the genetic background and immunopathogenic mechanisms of the autoantigens for the two ANCA types, MPO and PR3, there have been several attempts to classify AAV subtypes based on ANCA type as the primary criterion, rather than merely an aid to classification. This approach leads to three subtypes such as MPO-ANCA vasculitis, PR3-ANCA vasculitis, and ANCA-negative vasculitis (4). In 2017, the provisional criteria for GPA were proposed for distinguishing GPA from EGPA, and these criteria acknowledged the strong association between GPA and PR3-ANCA by assigning a highly positive score to an item of PR3-ANCA for the classification of GPA (6). Finally, the new classification criteria for AAV suggested by a joint group of the American College of Rheumatology and the European Alliance of the Associations for Rheumatology (the 2022 ACR/EULAR criteria) focused on the association between ANCA type and AAV subtype by assigning the highest positive scores for MPO-ANCA and PR3-ANCA when classifying for MPA and GPA, respectively, and assigning negative scores in the opposite cases (3, 7, 8).

Nevertheless, in real clinical practice, we uncommonly encounter patients classified as MPA or GPA in whom the opposite ANCA type is detected. This has raised the question of what effect the opposite ANCA type, in contrast to the associated ANCA type, could have on the initiation, exacerbation, and progression of the corresponding AAV subtype (9, 10). In this regard, PR3-ANCA positivity at diagnosis has been reported as a factor predictive of subsequent relapse of AAV to date, and of particular interest, it has also been suggested as a risk factor for the likelihood of relapse after rituximab administration (11–13). However, most studies have been conducted on the cohorts including both the whole patients with MPA and GPA patients, and there is a scarcity of representative studies that specifically define the clinical significance in only a population of patients with MPA directly unrelated to PR3-ANCA positivity at diagnosis. We wondered how PR3-ANCA positivity at diagnosis, which is not a major player in the process of the classification of MPA, might contribute to the clinical course of MPA. Hence, this study investigated the clinical utility of PR3-ANCA detected at diagnosis in MPA patients.

## 2 Materials and methods

### 2.1 Patients

We retrospectively analyzed the medical records of 337 Korean patients already classified as having AAV who had been enrolled in the Severance Hospital ANCA-associated VasculitidEs (SHAVE) cohort, and 191 patients with MPA who met the following inclusion criteria were included in this study and analyzed. The inclusion criteria for AAV of the SHAVE cohort were as follows: (i) the first diagnosis of AAV at this hospital by the specialized Rheumatologists; (ii) the fulfilment of the 2012 revised Chapel Hill Consensus Conference nomenclature of systemic vasculitides and the 2007 EMA algorithm (1, 2); (iii) the classification of MPA based on the 2022 ACR/EULAR criteria for MPA (7); (iv) no fulfilment of the 2022 ACR/EULAR criteria for GPA (8); (v) the presence of medical records sufficient for collecting clinical, laboratory, radiological, and histological data at diagnosis and during follow-up (7, 8). In principle, patients with missing data regarding baseline variables at diagnosis or outcomes/medications during follow-up were excluded from the SHAVE cohort; (vi) the presence of ANCA test results at diagnosis (within four weeks before and after diagnosis) (14); (vii) the follow-up for at least more than 6 months after diagnosis; (viii) no concurrent medical conditions affecting ANCA positivity (type) and AAV classification at diagnosis, such as malignancies, infectious diseases, and other inflammatory/autoimmune diseases (15, 16); (ix) no exposure to immunosuppressive drugs for AAV treatment or other medications affecting ANCA positivity within 4 weeks before diagnosis. This study was approved by the Institutional Review Board (IRB) of Severance Hospital, Seoul, Republic of Korea (IRB No. 4-2020-1071: approval date: 11 November, 2016) and was conducted in accordance with the principles of the Declaration of Helsinki. Owing to the retrospective design of the study and the use of anonymized patient data, the requirement for written informed consent was waived.

### 2.2 Clinical data at the time of diagnosis

At the time of diagnosis, demographic data (age, sex, body mass index, and smoking history) and comorbidities [type 2 diabetes mellitus (T2DM), hypertension, and dyslipidaemia] were collected. ANCA type and positivity were recorded, and AAV-specific indices, including the Birmingham Vasculitis Activity Score (BVAS) and the Five-Factor Score (FFS), were collected (17, 18). Laboratory results performed at the time of diagnosis, including erythrocyte sedimentation rate (ESR) and C-reactive protein (CRP), which reflect the burden of acute inflammation, were also recorded (19).

### 2.3 Clinical data during the follow-up period

During the follow-up period, this study investigated three major poor complications of MPA: subsequent all-cause mortality (ACM), relapse, and end-stage kidney disease (ESKD). ACM was defined as a death that occurred for any reason after the diagnosis (20). Subsequent relapse was defined as the re-emergence of MPA-related clinical symptoms and increased activity after achieving remission (21, 22). ESKD was defined as a medical state requiring kidney replacement therapy due to the deterioration of renal function. Patients who progressed to ESKD before diagnosis were not included as having a major complication of ESKD (23). For patients with each major complication of MPA, the follow-up period was defined as the duration from the diagnosis of MPA until the onset of the corresponding major complication. Conversely, for patients without each major complication, it was determined as the duration from MPA diagnosis to the last visit to the vasculitis clinic. Glucocorticoids and immunosuppressive drugs, which had been administered for MPA treatment after diagnosis, were also investigated.

### 2.4 ANCA measurement

Antineutrophil cytoplasmic antibody positivity was determined using two methods: the titers of MPO-ANCA and PR3-ANCA were quantified using an enzyme immunoassay, whereas perinuclear (P)-ANCA and cytoplasmic (C)-ANCA patterns were identified using an indirect immunofluorescent assay (14, 24). The results of ANCA measurements were assessed qualitatively (positive or negative) for analysis.

### 2.5 Interstitial lung disease

Interstitial lung disease (ILD) was defined and characterized according to radiological features, such as traction bronchiectasis, ground-glass opacities accompanied by traction bronchiectasis, reticulations alongside traction bronchiectasis, and honeycombing. ILD was diagnosed by both pulmonologists and independent radiologists based on chest imaging (HRCT) results. Traction bronchiectasis is an enlarged airway located at the periphery of the lungs. Ground-glass opacity denotes a cloudy appearance of the lungs while preserving the visibility of the vessels and airways. Reticulations are described in terms of their numerous linear densities. Honeycombing is characterized by a grouping of sub-pleural cysts, each measuring 3–10 mm in diameter and possessing clearly defined walls (25).

### 2.6 Statistical analysis

All statistical analyses were conducted using IBM SPSS Statistics for Windows, version 26 (IBM Corporation, Armonk, NY, United States). Continuous variables were expressed as the median with interquartile range. Categorical variables were presented as numbers and percentages. Differences between categorical variables were analyzed using the chi-square test or Fisher’s exact test, as appropriate. Meanwhile, differences between continuous variables were evaluated using the Mann-Whitney U test. Cumulative survival rates between groups were compared using the Kaplan-Meier survival test with the log-rank test. A *P*-value of less than 0.05 was considered statistically significant.

## 3 Results

### 3.1 Clinical data at the time of diagnosis

**FIGURE 1:**
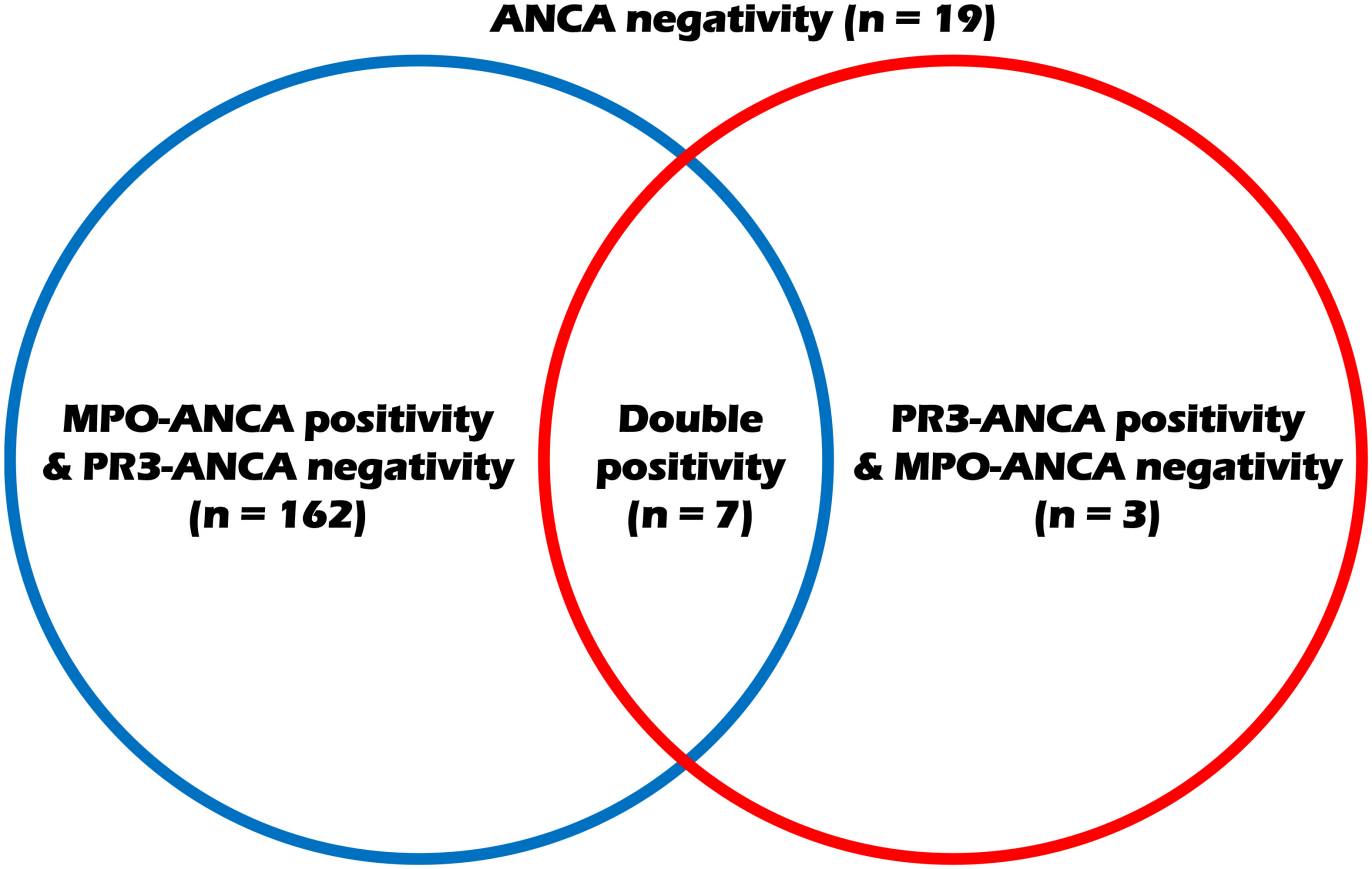
Disposition of the study patients according to the ANCA status. ANCA, antineutrophil cytoplasmic antibody; MPO, myeloperoxidase; PR3, proteinase 3.

Regarding variables at diagnosis, the median age of the 191 MPA patients was 64 years, with 68 males and 123 females. At the time of diagnosis, the most common major comorbidity was hypertension at 83%, followed by T2DM at 38%. Among 191 patients with MPA, 69 (88.5%) were positive for MPO-ANCA (P-ANCA), and PR3-ANCA was detected in only 10 (5.2%). All 10 patients who were positive for C-ANCA also had PR3-ANCA, and thus, PR3-ANCA (or C-ANCA) can be replaced with PR3-ANCA in this study. The ANCA status of the study patients are visualized in Figure 1. The medial BVAS, FFS, ESR, and CRP levels were 14.0, 2.0, 65.0 mm/hr, and 15.0 mg/L, respectively. The remaining data at diagnosis are presented in Table 1.

**TABLE 1:** Characteristics of patients with MPA (*N* = 191).

Variables	Values
**At the time of diagnosis**	
**Demographic data**	
Age (years)	64.0 (53.0−71.0)
Male sex [*N*, (%)]	68 (35.6)
Female sex [*N*, (%)]	123 (64.4)
BMI (kg/m^2^)	22.4 (20.4−24.6)
Ex-smoker	5 (2.6)
**Comorbidities [*N*, (%)]**	
T2DM	38 (19.9)
Hypertension	83 (43.5)
Dyslipidaemia	30 (15.7)
**ANCA type and positivity [*N*, (%)]**	
MPO-ANCA (or P-ANCA) positivity	169 (88.5)
PR3-ANCA (or C-ANCA) positivity	10 (5.2)
Both ANCAs	7 (3.7)
ANCA negativity	19 (9.9)
**AAV-specific indices**	
BVAS	14.0 (7.0−19.0)
FFS	2.0 (1.0−2.0)
**Laboratory results**	
White blood cell count (/mm^3^)	8,540.0 (6,230.0−12,170.0)
Hemoglobin (g/dL)	10.7 (9.2−12.7)
Platelet count (×1,000/mm^3^)	285.0 (222.0−387.0)
Fasting glucose (mg/dL)	101.0 (91.0−128.0)
Blood urea nitrogen (mg/dL)	20.1 (14.9−39.1)
Serum creatinine (mg/dL)	1.0 (0.7−2.2)
Serum total protein (g/dL)	6.8 (6.0−7.3)
Serum albumin (g/dL)	3.7 (3.1−4.2)
ESR (mm/hr)	65.0 (27.5−106.5)
CRP (mg/L)	15.0 (2.0−65.7)
**During the follow-up period**	
**Major complications**	
ACM [*N*, (%)]	32 (16.8)
Follow-up duration based on ACM (months)	52.0 (19.1−99.6)
Relapse [*N*, (%)]	43 (22.5)
Follow-up duration based on relapse (months)	30.6 (9.5−72.5)
ESKD [*N*, (%)]	39 (20.4)
Follow-up duration based on ESKD (months)	35.6 (8.8−86.6)
**Medications administered [*N*, (%)]**	
Glucocorticoid	173 (90.6)
Rituximab	40 (20.9)
Cyclophosphamide	98 (51.3)
Mycophenolate mofetil	59 (30.9)
Azathioprine	92 (48.2)
Tacrolimus	21 (11.0)
Methotrexate	20 (10.5)

Values are expressed as a median (25−75 percentile) or *N* (%). MPA, microscopic polyangiitis; BMI, body mass index; T2DM, type 2 diabetes mellitus; MPO, myeloperoxidase; ANCA, antineutrophil cytoplasmic antibody; P, perinuclear; PR3: proteinase 3; C, cytoplasmic; BVAS, the Birmingham vasculitis activity score; FFS, the five-factor score; ESR, erythrocyte sedimentation rate; CRP, C-reactive protein; ACM, all-cause mortality; ESKD, end-stage kidney disease.

### 3.2 Clinical data during the follow-up period

Among the 191 patients with MPA, 32 patients died over the course of the follow-up period. Meanwhile, 43 and 39 patients experienced subsequent relapse and progressed to ESKD, respectively. According to the records of drug administration associated with MPA treatment, 90.6% of patients received glucocorticoids, and the immunosuppressant with the highest frequency of administration was cyclophosphamide (51.3%), followed by azathioprine (48.2%) (Table 1).

### 3.3 Comparison of clinical data at the time of diagnosis

PR3-ANCA-positive MPA patients were significantly younger than PR3-ANCA-negative patients (45.5 vs. 64.0 years, *P* = 0.007). There was no significant difference in the positive detection rate of MPO-ANCA between patients with PR3-ANCA and those without. No significant differences in BVAS and FFS were found between the two groups, either. Among the laboratory results at diagnosis, white blood cell count, and blood urea nitrogen levels in PR3-ANCA-positive MPA patients were significantly lower than PR3-ANCA-negative patients (6,700 vs. 8,700/mm^3^, *P* = 0.035, and 13.8 and 20.4 mg/dL, *P* = 0.043, respectively). However, ESR and CRP levels did not differ between the two groups (Table 2).

**TABLE 2:** Comparative analysis between PR3-ANCA-positive MPA patients and PR3-ANCA-negative MPA patients.

Variables	PR3-ANCA-negative MPA patients (*N* = 181)	PR3-ANCA-positive MPA patients (*N* = 10)	*P*-value
**At diagnosis**			
**Demographic data**			
Age (years)	64.0 (54.0−72.0)	45.5 (21.5−64.3)	0.007
Male sex [*N*, (%)]	65 (35.9)	3 (30.0)	1.000
Female sex [*N*, (%)]	116 (64.1)	7 (70.0)	1.000
BMI (kg/m^2^)	22.4 (20.4−24.6)	22.1 (20.8−25.3)	0.732
Ex-smoker	5 (2.8)	0 (0)	1.000
**Comorbidities [*N*, (%)]**			
T2DM	35 (19.3)	3 (30.0)	0.420
Hypertension	80 (44.2)	3 (30.0)	0.518
Dyslipidaemia	29 (16.0)	1 (10.0)	1.000
**ANCA type and positivity [*N*, (%)]**			
MPO-ANCA (or P-ANCA) positivity	162 (89.5)	7 (70.0)	0.093
**AAV-specific indices**			
BVAS	14.0 (7.0−19.0)	12.0 (5.8−26.0)	0.958
FFS	2.0 (1.0−2.0)	1.0 (0−3.0)	0.471
**Laboratory results**			
White blood cell count (/mm^3^)	8,700.0 (6,235.0−12,390.0)	6,700.0 (5,602.5−7,415.0)	0.035
Hemoglobin (g/dL)	10.6 (9.1−12.7)	12.1 (9.9−14.4)	0.108
Platelet count (×1,000/mm^3^)	289.0 (218.5−391.0)	241.0 (223.3−287.8)	0.246
Fasting glucose (mg/dL)	102.0 (91.5−128.5)	91.0 (86.5−100.8)	0.078
Blood urea nitrogen (mg/dL)	20.4 (15.1−40.0)	13.8 (9.3−27.9)	0.043
Serum creatinine (mg/dL)	1.1 (0.7−2.4)	0.9 (0.6−1.4)	0.411
Serum total protein (g/dL)	6.8 (6.0−7.2)	7.7 (6.3−8.1)	0.085
Serum albumin (g/dL)	3.7 (3.1−4.1)	3.8 (3.6−4.4)	0.171
ESR (mm/hr)	69.0 (27.0−109.0)	40.5 (24.3−54.5)	0.108
CRP (mg/L)	16.0 (2.0−75.8)	10.5 (2.4−20.4)	0.267
**During the follow-up period**			
**Major complications**			
ACM [*N*, (%)]	31 (17.1)	1 (10.0)	1.000
Follow-up period based on ACM (months)	52.0 (18.1−95.4)	49.3 (22.5−154.1)	0.509
Relapse [*N*, (%)]	38 (21.0)	5 (50.0)	0.033
Follow-up period based on relapse (months)	31.1 (9.2−74.6)	24.9 (15.1−41.3)	0.397
ESKD [*N*, (%)]	38 (21.0)	1 (10.0)	0.690
Follow-up period based on ESKD (months)	35.3 (7.8−84.4)	49.3 (22.5−132.9)	0.273
**Medications administered [*N*, (%)]**			
Glucocorticoid	163 (90.1)	10 (100)	0.602
Rituximab	36 (19.9)	4 (40.0)	0.222
Cyclophosphamide	90 (49.7)	8 (80.0)	0.101
Mycophenolate mofetil	54 (29.8)	5 (50.0)	0.179
Azathioprine	85 (47.0)	7 (70.0)	0.201
Tacrolimus	19 (10.5)	2 (20.0)	0.302
Methotrexate	16 (8.8)	4 (40.0)	0.012

Values are expressed as a median (25–75 percentile) or *N* (%). Categorical variables were compared using chi-square test or Fisher’s exact test. Continuous variables were compared using the Mann-Whitney U test. PR3, proteinase 3; ANCA, antineutrophil cytoplasmic antibody; MPA: microscopic polyangiitis; BMI: body mass index; T2DM: type 2 diabetes mellitus; MPO, myeloperoxidase; P, perinuclear; C, cytoplasmic; BVAS, the Birmingham vasculitis activity score; FFS, the five-factor score; ESR, erythrocyte sedimentation rate; CRP, C-reactive protein; ACM, all-cause mortality; ESKD, end-stage kidney disease.

### 3.4 Comparison of clinical data during the follow-up period

Among the three major complications of MPA, the frequency of subsequent relapse in the PR3-ANCA-positive group was significantly higher than its frequency in the opposite group (50.0% vs. 21.0%, *P* = 0.033). Meanwhile, no differences in the frequencies of ACM and progression to ESKD were observed between the two groups. As a unique finding, on comparison of the administered medications, the frequency of MTX (methotrexate) use was determined to be remarkably elevated in patients belonging to the PR3-ANCA positive group compared to those in the PR3-ANCA negative group (40.0% vs. 8.8%, *P* = 0.012). However, it seemed difficult to draw a clinical significance because the total number of patients receiving MTX was too small to interpret (Table 2).

### 3.5 Cumulative relapse-free survival rates

**FIGURE 2:**
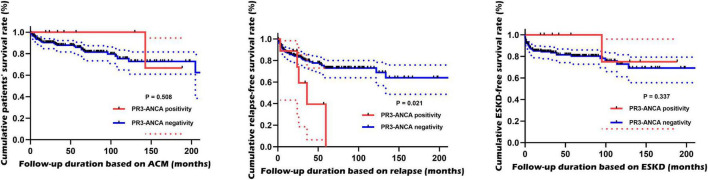
Comparison of cumulative survival rates. PR3-ANCA-positive MPA patients exhibited a significantly lower relapse-free survival rate than PR3-ANCA-negative MPA patients but not the other two major complications. PR3, proteinase 3; ANCA, antineutrophil cytoplasmic antibody; MPA, microscopic polyangiitis; ACM, all-cause mortality; ESKD, end-stage kidney disease.

Among the three major complications of MPA, PR3-ANCA-positive MPA patients exhibited a significantly lower relapse-free survival rate than PR3-ANCA-negative MPA patients (*P* = 0.021). Conversely, the incidence of the other two major complications did not differ between the two groups over the follow-up period (Figure 2).

## 4 Discussion

The 2022 ACR/EULAR criteria for MPA, which are currently used for the classification of MPA, assign +6 points to the item of MPO-ANCA positivity and −1 point to the item of PR3-ANCA positivity (7). This indicates that PR3-ANCA positivity at diagnosis makes a negative contribution to the classification of MPA, unlike MPO-ANCA positivity at diagnosis. Despite the negative influence of PR3-ANCA positivity upon MPA diagnosis (7), we occasionally encounter PR3-ANCA-positive MPA patients in real clinical settings. Here, we wondered whether PR3-ANCA positivity at diagnosis might be associated with the clinical characteristics of MPA alone, unlike its role in all subtypes of AAV (11–13), even though it is not directly involved in the process of MPA classification. However, a clear concept regarding this question has not yet been established. Therefore, in this study, we investigated the association between PR3-ANCA positivity at diagnosis and the course of the disease, particularly the occurrence of major complications, in MPA patients and obtained several interesting findings. First, at the time of diagnosis, PR3-ANCA-positive MPA patients were younger and had lower white blood cell count and blood urea nitrogen levels than PR3-ANCA-negative patients. Next, during the follow-up period, a higher frequency of subsequent relapse was observed in PR3-ANCA-positive MPA patients compared to PR3-ANCA-negative patients. Finally, during the follow-up period, PR3-ANCA-positive MPA patients exhibited a significantly lower relapse-free survival rate than PR3-ANCA-negative patients. Based on these results, we conclude that PR3-ANCA positivity at diagnosis is closely associated with subsequent relapse in MPA patients.

Meanwhile, a previous study demonstrated a significant association between PR3-ANCA positivity at diagnosis and subsequent relapse in EGPA patients (12), which could be inferred through the following hypothetic steps: first, the 2022 ACR/EULAR criteria for EGPA assign −3 points to the item of PR3-ANCA positivity and require a total score of 6 or more for EGPA classification (26); second, thus, patients who tested positive for PR3-ANCA at diagnosis should have met other criteria items by a three-point margin more than PR3-ANCA-negative patients for the classification of EGPA (26); and third, theoretically, the disease activity at diagnosis might have been higher, and the potential for relapse would have consequently been greater. By contrast, the 2022 ACR/EULAR criteria for MPA assign a high score of +6 points for the item of MPO-ANCA positivity and a relatively lower score of −1 point for the item of PR3-ANCA positivity, with a total score cut-off of five points (7). That is, the necessity to fulfill other criteria is relatively lower than the classification of EGPA, and consequently, the potential for relapse is inferred to be lower. Therefore, there might be a contradiction in directly applying the hypothesis from EGPA patients to MPA patients.

How could PR3-ANCA positivity at the time of diagnosis have contributed to subsequent relapse in MPA patients during the follow-up period? Based on these results, three theoretical inferences were attempted. The first inference was that subsequent relapse might have been affected by MPO-ANCA and PR3-ANCA double positivity rather than PR3-ANCA positivity alone. This line of inquiry was inspired by previous research demonstrating that double positivity of PR3-ANCA and anti-GBM contributes to subsequent relapse in patients with AAV, notwithstanding different antibody classes (27). To validate this inference, we reperformed a survival analysis for relapse by dividing the 191 patients into two groups based on MPO-ANCA and PR3-ANCA double positivity. Similar to the results in Figure 2, MPA patients with MPO-ANCA and PR3-ANCA double positivity showed a significantly lower cumulative relapse-free survival rate compared to those without (Supplementary Figure 1). Therefore, we carefully presume that the first inference regarding the effect of ANCA double positivity on subsequent relapse could be a possible explanation.

The second inference, conversely, was that PR3-ANCA positivity alone could be involved in the relapse process when the influence of MPO-ANCA was completely excluded. To validate this inference, the patients were divided into three groups: (i) PR3-ANCA negativity, (ii) PR3-ANCA positivity and MPO-ANCA positivity, and (iii) PR3-ANCA positivity and MPO-ANCA negativity. The cumulative incidence of relapse was relatively higher in both patients with MPO-ANCA and PR3-ANCA double positivity and those with PR3-ANCA positivity alone compared to patients with PR3-ANCA negativity, but there was no significant difference between the two groups (Supplementary Figure 2). Therefore, we speculate that the second inference regarding the effect of PR3-ANCA positivity alone on subsequent relapse might not be compelling.

The third inference was that the clinical symptoms related to relapse would have been the clinical symptoms associated with PR3-ANCA rather than MPO-ANCA. When analyzing 10 PR3-ANCA-positive MPA patients, none of the patients exhibited GPA surrogate markers at the time of diagnosis, which meant that none of the 10 patients could be classified as having GPA simultaneously. Five out of the 10 patients experienced relapse: four patients tested positive for both MPO-ANCA and PR3-ANCA at diagnosis, and the remaining one patient was only positive for PR3-ANCA at diagnosis. Among the four double-positive patients with relapse, who were positive for both MPO-ANCA and PR3-ANCA at diagnosis, two experienced both glomerulonephritis (GN) and interstitial lung disease (ILD) recurrence, and the remaining two had only ILD recurrence. Whereas one patient, who was positive for only PR3-ANCA at diagnosis, experienced recurrences of both GN and ILD at the same time (Supplementary Table 1). Therefore, given that ILD is suggestive of MPA and GN is not a specific manifestation for AAV subtype (7, 8), we conclude that the third inference regarding the possibility of relapse of clinical manifestations associated with PR3-ANCA is not valid.

A major strength of this study was that it focused on a pure MPA cohort, which minimized confounding from granulomatous disease and other AAV variants. Another major strength of this study was that the high quality and consistency of the data could be ensured by using the well-established SHAVE cohort, which enhanced the relevance of the findings, such as important outcomes. Additionally, it was believed that the novel observation linking PR3-ANCA positivity with ILD recurrence represented a potentially impactful contribution to the field.

### 4.1 Limitations

This study has several limitations. Firstly, due to the inherent limitations of a single-center study, this study did not include sufficient patients for the application of these results to real clinical practice immediately. This issue may provoke concerns about the enhanced susceptibility to type I error and the reduced generalizability. Another concern is that statistical analyses involving very low occurrence frequencies may limit the reliability and reproducibility of the statistics. Another limitation is the lack of multivariable analysis, which prevents meaningful adjustment for confounders such as age, ILD status, MPO-ANCA and PR3-ANCA double positivity, and treatment differences. Additionally, due to the unavoidable limitation of a retrospective study, it was not possible to adequately control the inner and outer various confounding variables. We realize that conclusions should be framed in terms of association rather than prediction or causality, and we believe the reason that the theoretical rationale outweighed the conclusions in this retrospective study may be ultimately attributable to the small number of patients and the low incidence rate. Nevertheless, the fact that a considerable number of patients selected from the largest and most systematically organized cohort in Korea were managed according to the same protocol could be expected to substantially overcome these limitations. We believe that a future prospective study with more patients will improve these limitations by multivariable analyses and provide more reliable and dynamic information on the clinical utility of PR3-ANCA positivity in patients who are newly diagnosed with MPA.

## 5 Conclusion

In conclusion, based on the results of the present study, we found that PR3-ANCA positivity at diagnosis is significantly associated with subsequent relapse in patients with MPA during follow-up. We believe that this study might provide several important clues to further studies investigating the clinical implications of the initial detection of PR3-ANCA in the disease course of MPA.

## Data Availability

The raw data supporting the conclusions of this article will be made available by the authors, without undue reservation.
