# Supplementary material for: Clinical utility of proteinase 3-antineutrophil cytoplasmic antibody at diagnosis in predicting subsequent relapse in patients with microscopic polyangiitis
Source: Front Med (Lausanne). 2026 Jan 12;12:1745280. doi: 10.3389/fmed.2025.1745280 (PMC12833265; doi:10.3389/fmed.2025.1745280)
Supplement: Supplementary file 1 [file Table_1.docx]

**Supplementary Table 1. In detail characteristics of ten PR3-ANCA-positive MPA patients**

| Order | sex | age | MPO-ANCA  Positivity  at diagnosis | PR3-ANCA  Positivity  at diagnosis | Items contributing to classifying MPA other than ANCA | Items leading to not classifying GPA other than ANCA | Medications | Relapsed clinical manifestations |
| --- | --- | --- | --- | --- | --- | --- | --- | --- |
| 1 | Female | 46 | Yes | Yes | GN, ILD | No GPA surrogate markers | GC, CYC, AZA, MTX | ILD |
| 2 | Female | 30 | Yes | Yes | GN, ILD | No GPA surrogate markers | GC, RTX, AZA | ILD |
| 3 | Female | 20 | Yes | Yes | GN, ILD | No GPA surrogate markers | GC, RTX, CYC, MMF, AZA, TAC, MTX | GN, ILD |
| 4 | Female | 20 | Yes | Yes | GN, ILD | No GPA surrogate markers | GC, RTX, CYC, MMF, AZA, TAC, MTX | GN, ILD |
| 5 | Male | 71 | Yes | Yes | GN, ILD | No GPA surrogate markers | GC, CYC, AZA |  |
| 6 | Male | 62 | Yes | Yes | GN, ILD | No GPA surrogate markers | GC, CYC, AZA |  |
| 7 | Male | 71 | Yes | Yes | GN, ILD | No GPA surrogate markers | GC, RTX, MMF |  |
| 8 | Female | 45 | No | Yes | GN, ILD | No GPA surrogate markers | GC, CYC, AZA, MTX | GN, ILD |
| 9 | Female | 22 | No | Yes | GN, ILD | No GPA surrogate markers | GC, CYC, MMF |  |
| 10 | Female | 58 | No | Yes | GN, ILD | No GPA surrogate markers | GC, CYC, MMF |  |

PR3: proteinase 3; ANCA: antineutrophil cytoplasmic antibody; MPA: microscopic polyangiitis; MPO: myeloperoxidase; GPA: granulomatosis with polyangiitis; GN: glomerulonephritis; ILD: interstitial lung disease, GC: glucocorticoids, CYC: cyclophosphamide, AZA: azathioprine, MTX: methotrexate; RTX: rituximab; MMF: mycophenolate mofetil, TAC: tacrolimus.
