# Supplementary figures and images for: Clinical utility of proteinase 3-antineutrophil cytoplasmic antibody at diagnosis in predicting subsequent relapse in patients with microscopic polyangiitis
Source: Front Med (Lausanne). 2026 Jan 12;12:1745280. doi: 10.3389/fmed.2025.1745280 (PMC12833265; doi:10.3389/fmed.2025.1745280)

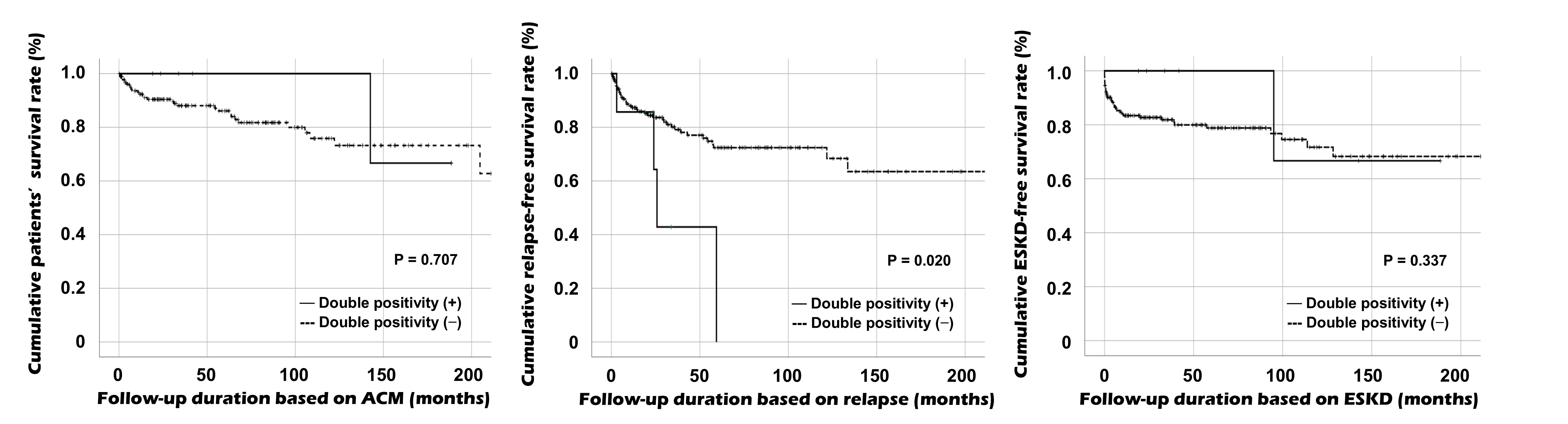

Supplement: Supplementary file 2 [file Image_1.tif]

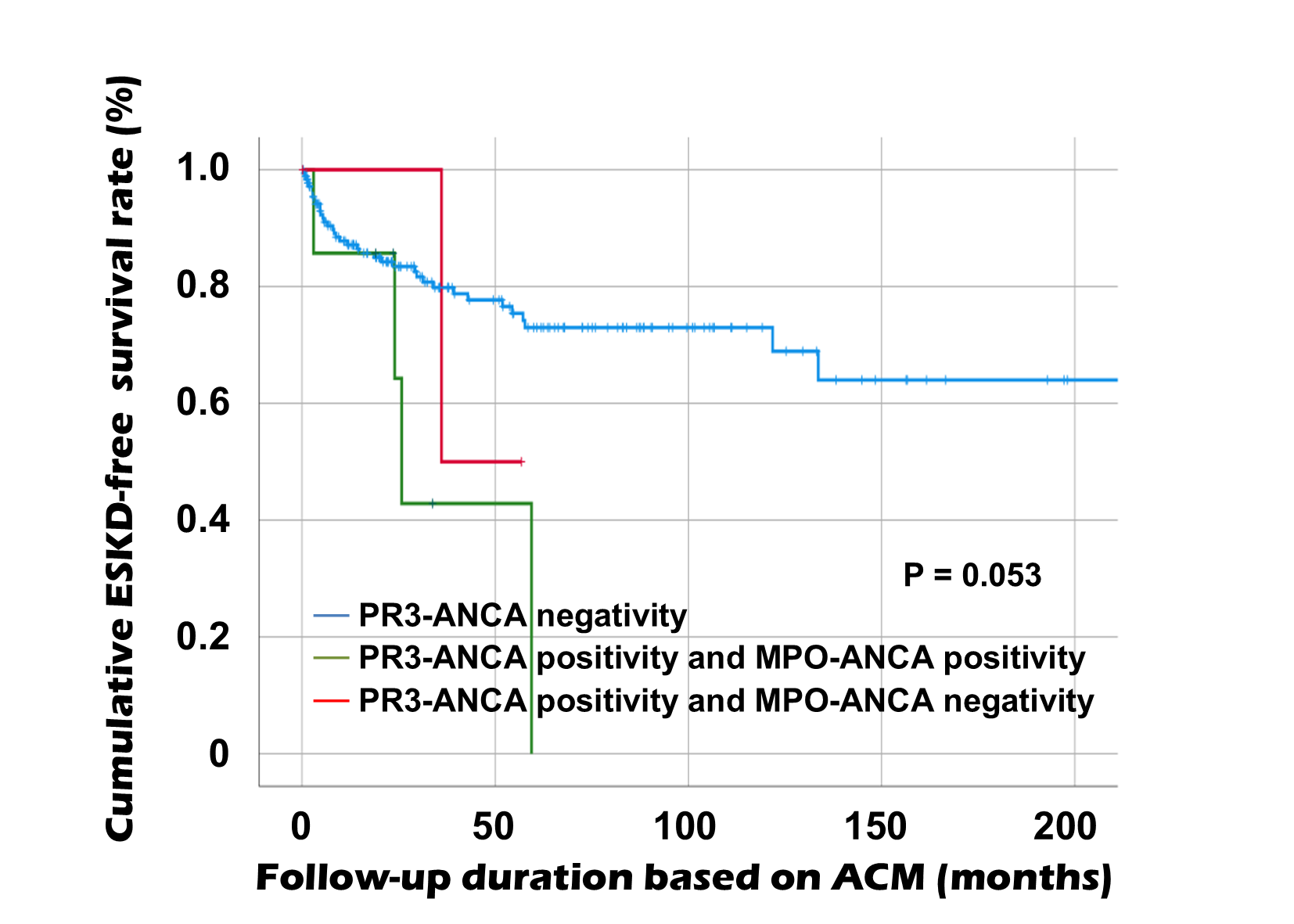

Supplement: Supplementary file 3 [file Image_2.tif]
